# Gastric Xanthoma Is Related to the Rapid Growth of Gastric Cancer

**DOI:** 10.3390/jcm10235704

**Published:** 2021-12-05

**Authors:** Ko Miura, Tadayuki Oshima, Akio Tamura, Ken Hara, Takuya Okugawa, Masashi Fukushima, Toshihiko Tomita, Hirokazu Fukui, Hiroto Miwa

**Affiliations:** Division of Gastroenterology and Hepatology, Department of Internal Medicine, Hyogo College of Medicine, Nishinomiya 663-8501, Japan; i.move.mountain@gmail.com (K.M.); akio@hyo-med.ac.jp (A.T.); k-hara@hyo-med.ac.jp (K.H.); okugawat@hyo-med.ac.jp (T.O.); ma-fukushima@hyo-med.ac.jp (M.F.); tomita@hyo-med.ac.jp (T.T.); hfukui@hyo-med.ac.jp (H.F.); miwahgi@hyo-med.ac.jp (H.M.)

**Keywords:** gastric cancer, growth, xanthomatosis

## Abstract

Early detection of gastric cancer is important. However, rapid growth of gastric cancers that cannot be resected endoscopically occurs even with periodic check-ups. Accordingly, we assessed factors associated with the speed of gastric cancer growth by examining historical endoscopic images. A total of 1996 gastric cancer cases were screened, and characteristics of lesions with slow and rapid growth were assessed. A total of 114 lesions from 114 patients were included in the assessment. Sixty slow-growing and fifty-four rapidly growing gastric cancers were compared. Female sex and incidence of lesions in the lower part of the stomach were significantly less frequent in the rapid-growth group than in the slow-growth group. History of endoscopic treatment tended to be more frequent in the rapid-growth group. Age, body mass index, histology, *Helicobacter pylori* status, and medications did not differ significantly between groups. Xanthoma was significantly related to rapid growth of gastric cancer, and map-like redness tended to be more frequent in the rapid-growth group in univariate analysis. Xanthoma was significantly related to rapid growth of gastric cancer on multivariate analysis. Further studies are warranted to clarify the pathophysiological mechanisms involved in the speed of gastric cancer growth.

## 1. Introduction

Gastric cancer remains an important cancer worldwide, responsible for over 1,000,000 new cases and an estimated 783,000 deaths in 2018 (equivalent to 1 in every 12 deaths globally), making it the fifth most frequently diagnosed cancer and the third leading cause of cancer death [1]. On a global scale, the incidence of gastric cancer is higher in Asia than in Europe or Northern America. In a previous report, cases of *Helicobacter pylori* gastritis from seven countries were evaluated and the highest scores for antral atrophy were found in Japan [2]. Chronic atrophic gastritis is strongly associated with gastric cancer, and the incidence of gastric cancer is high in East Asia [3].

Early detection of gastric cancer has therefore recently been the focus of screening examinations. Subjects in Korea who underwent upper gastrointestinal endoscopy were less likely to die from gastric cancer [4]. Upper gastrointestinal endoscopy screening achieved a 30% reduction in gastric cancer mortality within 36 months before the date of gastric cancer diagnosis [5]. Based on such data, health check-ups by upper gastrointestinal endoscopy are currently recommended every other year in Japan and Korea [6]. When cancers are detected at an early stage and can be treated endoscopically, quality of life is significantly better after endoscopic submucosal resection compared to surgery [7].

However, rapid growth of gastric cancers that could not be resected endoscopically has been reported even in patients receiving periodic check-ups [8]. Previous reports have shown the natural history of gastric cancers in specific subjects who were not treated for various reasons [9]. A report showed that the median time to progression from tumor-node-metastasis stage I to stage II was 34 months, and no risk factors for cancer progression were found [10]. Cases of gastric cancer remained as mucosal cancer for 3–9 years [11,12]. As cancers that remain untreated necessarily show specific conditions and the patients are often not in a good state of health, outcomes from these patients cannot be generalized as representing the natural history of gastric cancer. Furthermore, no studies have assessed the speed of gastric cancer growth and prospective follow-up of cases is ethically unacceptable.

Studies assessing patients and endoscopic factors (e.g., sex, gastric xanthoma, atrophy, and intestinal metaplasia) associated with the speed of gastric cancer growth are therefore needed to reveal those high-risk gastric cancer patients who should be followed closely. Accordingly, the present study was conducted to identify factors associated with the speed of gastric cancer growth by examining historical images from endoscopy.

## 2. Materials and Methods

### 2.1. Study Design, Setting and Participants

Patients who underwent esophagogastroduodenoscopy (EGD) at Hyogo College of Medicine (Nishinomiya, Japan) from March 2012 to December 2019 were assessed. During the study period, 49,587 EGDs were performed, and 1996 cases of gastric cancer were diagnosed. After excluding cases with no history of EGD 1 year or more before detection, cases and lesions with a history of EGD were counted.

**Figure 1:**
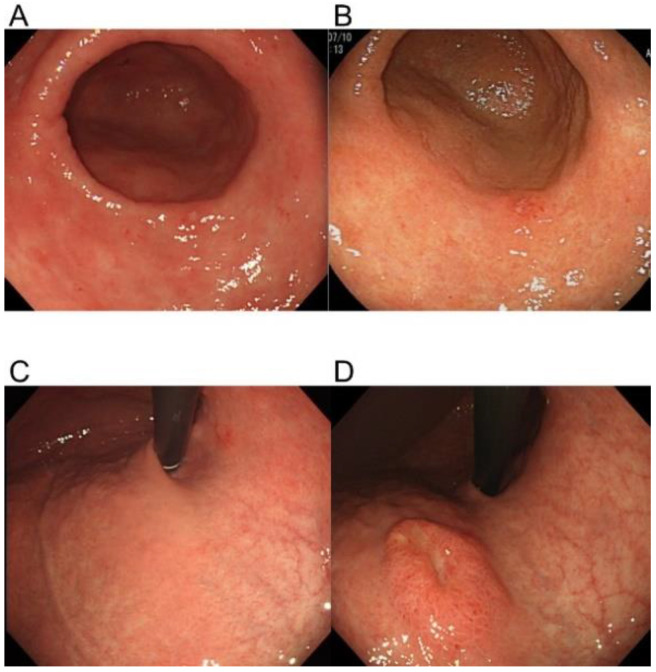
Representative lesions showing slow and rapid gastric cancer growth. A slow-growing lesion at two years before diagnosis (**A**) and at the time of diagnosis (**B**). The site of a rapidly growing lesion at two years before diagnosis (**C**) and at the time of diagnosis (**D**).

Patients were excluded if they satisfied any of the following exclusion criteria: remnant stomach, familial adenomatous polyposis (FAP), or lack of data years before detection (including cases without identified lesion location in the initial EGD). Included cases were classified as a slow-growth group, comprising patients whose findings showed cancer in previous endoscopic images (Figure 1), or a rapid-growth group, comprising cases whose findings showed no cancer in previous endoscopic images (Figure 1) [6,13,14]. The study protocol was approved by the ethics committee/institutional review board at Hyogo College of Medicine, Japan (approval no. 201909-047) on 24 June 2019.

### 2.2. Definition of Lesion

The date of EGD examination that led to the diagnosis of gastric cancer was used as the index data for endoscopy. The slow-growth group comprised cases in which the lesion was detectable with careful retrospective inspection at least 2 years before the index EGD examination as initial data [8]. We used a period of 2 years because the current recommended interval for screening upper gastrointestinal endoscopy for health check-ups is every other year [6]. The color and surface structure observed during the initial EGD examination were compared between the expected location and surrounding mucosa using saved images, and lesions were defined as present when findings were different from those of the surrounding mucosa. The rapid-growth group comprised cases in which no lesions were detected 1–2 years before the index EGD.

**Figure 2:**
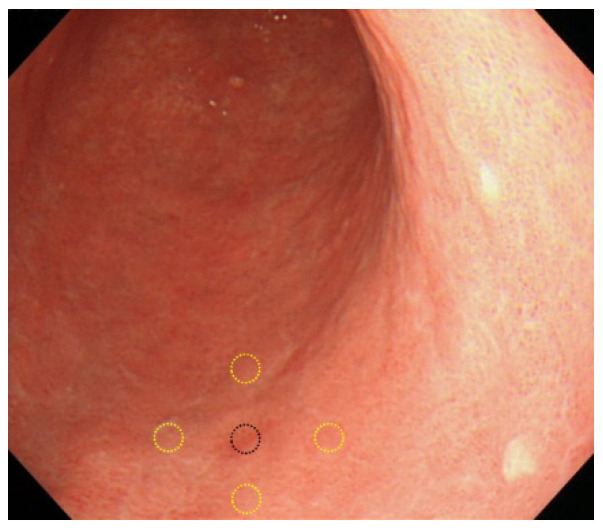
Assessment of color index. One central lesion and 4 surrounding points were evaluated to determine the color index. Black circle, lesion area. Yellow circles, surrounding areas.

The definition of no lesion at two years before the index EGD was dependent on the review of the two experts. To make the decision more objective, differences in color on images, as one of the factors used to distinguish lesions, were assessed using image-analysis software (ImageJ; National Institutes of Health, Bethesda, MD, USA). The method for evaluating objectivity was based on a previously reported technique [15]. Briefly, endoscopic images were converted into JPEG images. Using the default method, i.e., the “Measure” tool in the “Analyze” menu, mean gray values (MGVs) of the lesion and perilesional normal mucosa were calculated. MGV of the lesion was measured at one location, and the perilesional mucosa was calculated as the average of MGVs from four locations. Color index was defined using the following equation: color index = |(lesion MGV/mean perilesional MGV) − 1|. With this method, an absence of any color difference between the lesion and perilesional mucosa would provide a value of 0 (Figure 2).

### 2.3. Definition of Related Factors

Factors were identified by comparing the slow- and rapid-growth groups. Factors used in the present study were the characteristics of the patient and lesion, including age, sex, body mass index (BMI), family history of gastric cancer, history of endoscopic treatment, location, size, macroscopic type [16], histology, *H. pylori* infection, use of proton pump inhibitors (PPIs), use of statins, use of steroids, use of aspirin, and use of non-steroidal anti-inflammatory drugs (NSAIDs). The characteristics of endoscopic findings were defined using the Kyoto classification of gastritis [17] and confirmed by index endoscopies. Cancer location was classified into two types: lower part (angle and antrum) and other parts. *H. pylori* infection was detected based on endoscopic features, rapid urease test, urea breath test, or serum antibody. The use of PPIs, statins, steroids, aspirin, or NSAIDs was defined when these factors were present for more than 6 months before the detection of gastric cancer.

### 2.4. Statistical Analysis

To determine factors related to the rapid growth of gastric cancer, we estimated odds ratios and 95% confidence intervals (CI). Age and BMI were compared using Student’s *t*-test, and macroscopic type was compared using Fisher’s exact test. Sex, family history of gastric cancer, history of endoscopic treatment, location, histology, *H. pylori* infection, PPI, statin, steroid, aspirin, NSAID, atrophy, xanthoma, map-like redness, intestinal metaplasia, diffuse redness, enlarged fold and nodularity were compared using the chi-square test for univariate analyses and unconditional logistic regression for multivariate analysis. Factors showing values of *p* < 0.1 on univariate analysis were used in the multivariate analysis. All reported *p*-values were two-sided, and values less than 0.05 were considered statistically significant. JMP^®^ was used for all statistical analyses (version 14; SAS Institute Inc., Cary, NC, USA).

## 3. Results

### 3.1. Characteristics of Patients and Lesions

**Figure 3:**
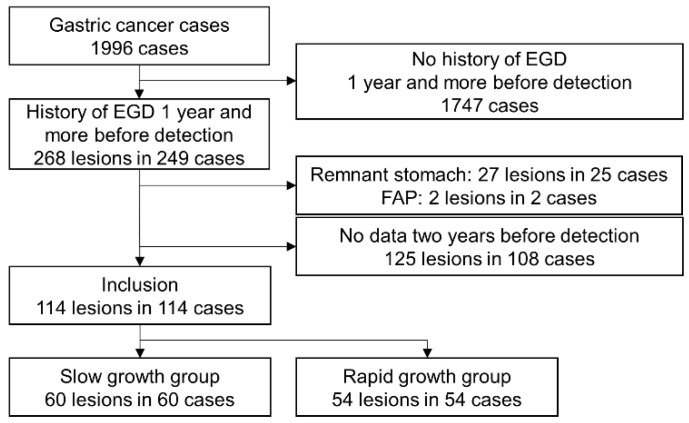
Patient flowchart. EGD, esophagogastroduodenoscopy; FAP, familial adenomatous polyposis.

During the study period, 49,587 EGDs were performed, and 1996 cases were diagnosed with gastric cancer. Patients were excluded if they satisfied any of the following exclusion criteria: no history of EGD ≥ 1 year before detection (*n* = 1747), remnant stomach (*n* = 25), FAP (*n* = 2), or no data 2 years before detection (*n* = 108). A total of 114 lesions from 114 patients were finally included in the present study (Figure 3). The mean age of patients was 72.1 years, and 42 patients were female. Three cases were not infected with *H. pylori*, 19 showed present infection, and 92 were post-eradication. The pathological diagnoses of the slow-growth group obtained from index data were pap, 1; tub1, 47; tub2, 4; por1, 2; por2, 2; sig, 2; and gastric adenocarcinoma of fundic gland type (GA-FG), 2. The pathological diagnoses of the rapid-growth group were pap, 2; tub1, 38; tub2, 5; por1, 1; por2, 4; sig, 2; and GA-FG, 2. Gastric xanthoma was detected in 53 (46.5%) of the 114 patients included in the present study. Reasons for undergoing EGD were periodic check-up due to *H. pylori* infection and eradication history in 97.4%.

### 3.2. Factors Related to Speed of Gastric Cancer Progression

Data for the initial endoscopy were extracted at 41 months (interquartile range (IQR), 29–64 months) before the index endoscopy for the slow-growth group and at 16 months (IQR, 13–24 months) for the rapid-growth group. To make identifying the presence of the initial lesion more objective, differences in color were assessed by color index in the areas of lesions. Color index was significantly higher in the slow-growth group than in the rapid-growth group (0.16, 95% CI 0.11–0.21 vs. 0.04, 95% CI 0.03–0.06, respectively; *p* < 0.01).

The demographics and characteristics of the slow-growth group (*n* = 60) and rapid-growth group (*n* = 54) are summarized in Table 1. Female sex and presence of the lesion in the lower stomach were significantly less frequent in the rapid-growth group than in the slow-growth group (Table 1). History of endoscopic treatment tended to be more frequent in the rapid-growth group, although the difference was not significant. Age, BMI, histology of the lesion, *H. pylori* infectious status, and use of medications likewise did not differ between groups. In cases after *H. pylori* eradication, the duration until the detection of gastric cancer after eradication did not differ between groups. Lesion depth did not differ between slow- and rapid-growth groups (slow growth: m, 51; sm1, 5; sm2, 2; and ss, 2 vs. rapid growth: m, 43; sm1, 1; sm2, 5; mp, 1; and ss, 4).

Univariate analyses of characteristics from endoscopic images showed that xanthoma correlated significantly with rapid growth of gastric cancer (Table 2). The incidence of map-like redness tended to be greater in the rapid-growth group, although the difference was not significant. Atrophy, intestinal metaplasia, and diffuse redness did not differ between groups. Multivariate analysis showed that xanthoma was significantly related to rapid growth of gastric cancer (Table 3).

## 4. Discussion

The present study assessed the speed of gastric cancer growth by assessing previous upper gastrointestinal endoscopic images to reveal characteristics of rapidly progressing cancers that were found by surveillance approaches. Xanthoma was found to be associated with rapid growth of gastric cancer. Although the natural history of gastric cancer was first assessed 65 years ago [18], no reports appear to have described factors related to the speed of gastric cancer growth. The present study is the first to reveal factors affecting the speed of gastric cancer progression.

### 4.1. H. pylori Infection

*H. pylori* infection could be one factor related to the speed of gastric cancer growth, as *H. pylori* itself causes chronic inflammation and acts to promote stomach carcinogenesis [19], while eradication of *H. pylori* infection reduces the incidence of gastric cancer [20]. However, this infection was not associated with the speed of gastric cancer growth in the present study. Therefore, speed of gastric cancer growth may be unrelated to active *H. pylori* infection. Recent studies have shown that map-like redness represents a risk factor for the development of gastric cancer after successful eradication of *H. pylori* [21,22]. However, besides xanthoma, these endoscopic characteristics were not associated with the speed of gastric cancer growth in the present study.

### 4.2. Gastric Xanthoma

Gastric xanthoma, also known as xanthelasma or lipid island, is a small, yellowish-white plaque or nodule characterized by accumulation of lipid, including oxidized low-density lipoprotein, in histiocytic foam cells [23,24]. The incidence of gastric xanthomas varies from 0.23% to 7% [25,26]. Previous reports have indicated that the presence of gastric xanthoma and the incidence of gastric cancer are significantly associated [27,28], and gastric xanthoma offers a predictive marker for metachronous and synchronous gastric cancer [29]. However, no reports have clarified the relationship to the speed of gastric cancer progression. Kaiserling et al. reported that increased release of oxygen free radicals may be involved in the formation of gastric xanthoma [24]. Oxygen free radicals are well known to cause DNA damage and to play roles in the pathogenesis of various malignancies [30]. Such mechanisms of xanthoma might also be related to the speed of gastric cancer progression. The present data showed that xanthoma was independently related to the speed of gastric cancer growth after adjustment by sex. Although some reports have described a male predominance in gastric xanthoma [31,32], the data remain conflicting [25,26,27]. Further studies are warranted to clarify the relationship between xanthoma and the speed of gastric cancer progression.

### 4.3. Histological Type

Interestingly, histological types were not associated with speed of lesion growth in the present study, although diffuse-type gastric cancer was speculated to be more common in the rapid-growth group. Multivariate analysis indicated that among pathological variables of the tumor, a histological type of poorly differentiated gastric cancer was one independent prognostic factor [33]. As all new lesions that were not apparent in the index examination were defined as rapidly growing lesions in the present study, newly identified slow-progressing cancers might have also been included in the rapid-growth group. Furthermore, signet-ring cells confer favorable prognosis in the early stage [34,35]. These factors might have contributed to the lack of significant differences in histological types between the two groups.

### 4.4. Screening Examination

Recently, although screening examinations have been performed to identify various cancers in the early stages, reports have indicated that small cancers found by screening examinations do not affect the overall survival of patients [36,37,38]. Therefore, to prevent overdiagnosis and overtreatment, there is a movement to avoid categorizing lesions that do not affect survival as cancers [37]. Conversely, gastric cancers that progress rapidly exist, and a background mucosa with xanthoma is one factor related to rapid growth. As surgical resection can negatively affect the quality of life of patients, early detection of lesions that have potential to grow rapidly is of great significance. Strict follow-up and detailed observation are needed to detect lesions in the early stage among such high-risk patients.

### 4.5. Limitations

Several limitations to the present study must be considered. First, the study was retrospective, although a prospective study to follow cancer progression would be ethically unworkable. Second, data were extracted from one tertiary care center, so various selection biases would be present, and some patients have a history of endoscopic treatment or other concomitant diseases. The initial EGD may have been for reasons other than screening for cancers in some cases. These factors were therefore also assessed in this analysis, revealing no correlations. Third, the rapid-growth group might have also included some newly identified slow-growing lesions, because the rapid growth in the present study only meant no lesion for at least 1 year before detection. Some factors not identified as significant in the multivariate analysis of the present study might still be candidates for affecting the speed of gastric cancer growth. Fourth, xanthoma would not be the only factor affecting the speed of gastric cancer growth. Examination of genetic, genomic alteration, or DNA methylation in the background gastric mucosa might reveal biomarkers related to rapid cancer growth.

## 5. Conclusions

Xanthoma was identified as a factor related to the rapid growth of gastric cancer. As prospective studies evaluating the natural history of gastric cancer cannot be performed due to ethical problems, the present study revealed a potentially important clinical factor that affects the speed of gastric cancer growth. Further studies are warranted to reveal the pathophysiological mechanisms involved in the speed of gastric cancer growth.

## Figures and Tables

**Table 1:** Characteristics of patients and lesions.

	Slow Growth (*n* = 60)	Rapid Growth (*n* = 54)	Univariate OR (95% CI)	*p*-Value
Age, mean (SD), years	72.4 (10.1)	71.9 (7.8)	0.99 (0.95–1.04)	0.785
Sex (female), *n* (%)	28 (46.7)	14 (25.9)	0.40 (0.18–0.88)	0.023
BMI, mean (SD)	22.9 (3.6)	23.4 (3.6)	1.04 (0.94–1.16)	0.439
Endoscopy interval, months, median (IQR)	41 (29–64)	16 (13–24)	0.65 (0.52–0.81)	<0.001
Family history of GC, *n* (%)	1 (1.7)	1 (1.9)	1.11 (0.07–18.2)	0.940
History of endoscopic treatment, *n* (%)	12 (20.0)	20 (37.0)	2.04 (0.89–4.66)	0.092
Location (lower part), *n* (%)	38 (63.3)	22 (40.7)	0.40 (0.19–0.85)	0.017
Size, median (IQR), mm	12 (5.3–15.8)	10 (6.0–16.0)	0.98 (0.95–1.02)	0.274
Macroscopic type, *n* (%)				
0–I	3 (5.0)	0		0.246 ^†^
0–IIa	19 (31.7)	14 (25.9)		0.540 ^†^
0–IIc	32 (43.3)	34 (57.4)		0.345 ^†^
0–IIa + IIc	4 (6.7)	1 (1.9)		0.367 ^†^
Type 1	1 (1.7)	0		1.000 ^†^
Type 2	0	4 (7.4)		0.253 ^†^
Type 3	1 (1.7)	0		1.000 ^†^
Type 4	0	0		-
Type 5	0	1 (1.9)		0.473 ^†^
Histology (diffuse), *n* (%)	6 (10.0)	7 (13.0)	1.34 (0.42–4.27)	0.620
*H. pylori* infection, *n* (%)				
Negative	3 (5.0)	0 (0.0)	-	-
Eradication	44 (73.3)	48 (88.9)	1.00	-
Positive	13 (21.7)	6 (11.1)	0.42 (0.15–1.21)	0.108
PPI, *n* (%)	29 (48.3)	18 (33.3)	0.53 (0.25–1.14)	0.106
Statin, *n* (%)	23 (38.3)	14 (25.9)	0.56 (0.25–1.25)	0.160
Steroid, *n* (%)	7 (11.7)	10 (18.5)	1.72(0.60–4.89)	0.309
Aspirin, *n* (%)	8 (13.3)	5 (9.3)	0.66 (0.20–2.17)	0.497
NSAID, *n* (%)	7 (11.7)	2 (3.7)	0.29 (0.06–1.47)	0.135

^†^ Fisher’s exact test. OR, odds ration; SD, standard deviation; BMI, body mass index; GC, gastric cancer; IQR, interquartile range; PPI, proton pump inhibitor; NSAID, non-steroidal anti-inflammatory drug.

**Table 2:** Characteristics of endoscopic image.

	Slow Growth (*n* = 60)	Rapid Growth (*n* = 54)	Univariate OR (95% CI)	*p*-Value
Atrophy (open), *n* (%)	48 (80.0)	46 (85.2)	0.70 (0.26–1.86)	0.469
Xanthoma, *n* (%)	22 (36.7)	31 (57.4)	2.33 (1.10–4.94)	0.028
Map-like redness, *n* (%)	8 (13.3)	15 (27.8)	2.50 (0.96–6.49)	0.060
Intestinal metaplasia, *n* (%)	51 (85.0)	40 (74.1)	0.50 (0.20–1.28)	0.151
Diffuse redness, *n* (%)	11 (18.3)	8 (14.8)	0.77 (0.29–2.10)	0.615
Enlarged fold, *n* (%)	2 (3.3)	0 (0.0)	-	-
Nodularity, *n* (%)	0 (0.0)	0 (0.0)	-	-

OR, odds ratio; CI, confidence interval.

**Table 3:** Multivariate analysis.

	Multivariate OR (95% CI)	*p*-Value
Sex (female)	0.49 (0.21–1.16)	0.103
Location (lower part)	0.53 (0.24–1.20)	0.129
History of endoscopic treatment	1.26 (0.50–3.14)	0.627
Xanthoma	2.39 (1.06–5.39)	0.037
Map-like redness	1.96 (0.69–5.54)	0.207

OR, odds ratio.

## Data Availability

The data presented in this study are available on request from the corresponding author. The data are not publicly available due to ethical restrictions.
